# Time and spatial trends in lymphoid leukemia and lymphoma incidence and survival among children and adolescents in Manitoba, Canada: 1984-2013

**DOI:** 10.1371/journal.pone.0175701

**Published:** 2017-04-21

**Authors:** Xibiao Ye, Mahmoud Torabi, Lisa M. Lix, Salaheddin M. Mahmud

**Affiliations:** 1 Department of Community Health Sciences, Max Rady College of Medicine, Rady Faculty of Health Sciences, University of Manitoba, Winnipeg, Manitoba, Canada; 2 George & Fay Yee Centre for Healthcare Innovation, University of Manitoba, Winnipeg, Manitoba, Canada; UNM Cancer Center, UNITED STATES

## Abstract

**Objectives:**

To test for time and spatial trends in lymphoid malignancies, including lymphoid leukemia (LL), Hodgkin lymphoma (HL), and non-Hodgkin lymphoma (NHL), in children and adolescents in the province of Manitoba, Canada.

**Methods:**

Incident cases diagnosed between 1984 and 2013 were identified from the Manitoba Cancer Registry. We assessed time trends in age-standardized incidence rates using joinpoint regression and in 5-year relative survival using Poisson regression model. Kulldorff's scan method was used to assess spatial variation and clustering.

**Results:**

Age-standardized incidence rates (per million person-years) in males and females were 34.0 (95% confidence interval [CI] 28.9–39.1) and 26.2 (95% CI 21.5–30.7) for LL, 10.5 (95% CI 7.7–13.3) and 12.5 (95% CI 9.4–15.7) for HL, 12.5 (95% CI 9.3–15.4) and 7.7 (95% CI 5.2–10.2) for NHL (except for Burkitt lymphomas), and 3.2 (95% CI 1.6–4.7) and 1.5 (95% CI 0.4–2.5) for Burkitt lymphomas. Age- and sex- standardized LL incidence rate increased 1.4% (95% CI 0.3%-2.5%) per year, while the changes for HL and NHL incidence rates were not statistically significant. There were geographic differences in age-standardized incidence rates for LL, HL, and NHL and spatial clusters were detected in southern part of the province. Five-year relative survival has improved over time and there was no difference between rural and urban areas.

**Conclusions:**

Lymphoid leukemia incidence rate increased over time and varied by geographic area. Further research should examine the factors contributing to these trends.

## Introduction

Hematological malignancies as a group are the most common cancer in children (under age of 15 years) and adolescents (15–19 years) and account for 40% of total cancer cases in this population [1]. Acute lymphoid leukemia (ALL) is the most common hematological malignancy, followed by Hodgkin lymphoma (HL) and non-Hodgkin lymphoma (NHL) [1]. Little is known about the etiology of the cancers and there are only a few established risk factors, including high-level ionizing radiation [2], certain chemotherapeutic agents [3] (e.g., diethylstilbestrol [4]), certain genetic disorders (e.g., Down syndrome [5]), and congenital immunodeficiency diseases [1].

Analyzing time and spatial trends of cancer incidence may provide useful information to generate new hypotheses for etiological research. Despite extensive research on time trends in hematological malignancy incidence in adults, few studies have been conducted in children and adolescents. A recent international comparative analysis showed differences in temporal trends in leukemia and lymphoma incidence rates in children by country [6]. Data have also shown geographical variations in incidence among children and adolescents at the country level [7–11], but less is known about the variation within smaller areas (e.g., provinces or regions). Short-term and long-term survival of hematological malignancy patients have been improved over time [1,7,10], but disparities related to factors such as ethnicity and geography have been shown [12–14]. In this article, we present data on incidence and survival and their time and spatial trends for lymphoid malignancies in children (under 15 years) and adolescents (15 to 19 years) in the province of Manitoba, Canada.

## Materials and methods

### Data sources

Cancer diagnosis information was retrieved from the Manitoba Cancer Registry (MCR), a population-based registry operated by CancerCare Manitoba (CCMB). Cancer patients were originally coded using different editions of the International Classification of Disease for Oncology (ICD-O) and were converted to the 3rd edition (ICD-O-3) [15]. Reporting of cancer cases to the MCR is mandatory and MCR is regularly audited by the North American Association of Central Cancer Registries [16]. The quality of cancer registry data has been consistently very high. Most cases are pathologically-confirmed (94% for cases registered between 2006 and 2010) and less than 2% of registrations originate from death certificates [16].

Histology and topography codes were used to identify lymphoid leukemia (ICD-O-3 histology codes 9835–9836), Hodgkin lymphoma (ICD-O-3 histology codes 9650–9655, 9659, 9661–9665, 9667), and non-Hodgkin lymphoma (ICD-O-3 histology codes 9590–9591, 9596, 9670–9671, 9673, 9675, 9678–9680, 9684, 9687, 9688–9691, 9695, 9698–9702, 9705, 9708–9709, 9714, 9716–9719, 9724–9729, 9731–9735, 9737–9738, 9760–9762, 9764–9769, 9970; 9811–9818, 9837, 9823, and 9827 when C000-C419, C422-C423, C425-C809 as ICD-O-3 site). Cases were then classified using the International Classification of Childhood Cancer, 3rd Edition (ICCC-3) [17].

Other patient characteristics including sex, age at diagnosis, year of diagnosis, residential location at the time of diagnosis (i.e., 6-digit postal code) were also obtained from the MCR. All child and adolescent cases registered with MCR between 1984 and 2013 were included. Household income quintile at diagnosis was determined based on dissemination area level average household income derived from Canadian Census data [18]. Manitoba population counts by age, sex, and year were obtained from the Manitoba Health Insurance Registry. This research was approved by the University of Manitoba Research Ethics Board, Manitoba Health Information Privacy Committee, and CancerCare Manitoba Research Resource Impact Committee.

### Statistical analysis

The total number of incident cases were described by sex and subtype. Sex ratios and median age of diagnosis were also calculated. Age was grouped using 5-year intervals (0–4, 5–9, 10–14, 15–19 years). Annual age-standardized incidence rates were calculated using the 2006 Canadian population as the standard population. Age-specific incidence rates were also calculated. We tested the time trend in incidence using the Joinpoint Regression Program [19]. The joinpoint regression method first tested the trend with no jointpoint (i.e., linear model) and then determined whether more joinpoints (up to 3) need to be added, based on Permutation Test and Bayesian Information Criterion [20]. Annual percentage change (APC) and 95% confidence intervals (CIs) were estimated [20].

In Manitoba, regional health authorities (RHAs, 5 in total as of 2013) are responsible for the delivery and administration of health services in a specific geographical area. Each RHA is divided into smaller geographical regions. These include 25 neighborhood clusters in the provincial capital Winnipeg, with an average of 6,500 children and adolescents in each cluster as of July 1, 2013. As well, there are 72 districts, with an average of 2,300 children and adolescents in each district, outside of Winnipeg. For the spatial analysis, we first assigned each case to a single district or neighborhood cluster according to the 6-digit postal code for the patient’s place of residence at the date of diagnosis. We used an empirical Bayes method to estimate smoothed incidence rate ratios by geographical area, adjusting for sex, age, and average household income at diagnosis (area-based income measurement) [21]. Ratios for districts were calculated using the incidence for the entire province as the reference; ratios for Winnipeg neighborhood clusters were calculated using Winnipeg incidence rate as the reference. Kulldorff's scan method was used for cluster detection [22,23]. The most likely cluster, i.e., the cluster that is least likely to be due to chance is identified. All spatial analyses were conducted using R package “SpatialEpi (version 1.2.1)” [24].

We calculated 5-year relative survival, the ratio of observed survival of cancer patients to the expected survival of a comparable Canadian general population assumed free of cancer, using the period analysis method [25]. Expected survival was calculated based on Canadian age- and sex- specific mortality by year provided by the Human Mortality Database (www.mortality.org/), according to the Ederer II method [26]. Standard errors for relative survival were calculated using the Greenwood method and 95% confidence intervals were derived using logarithmic transformation [27]. A Poisson regression model was used to test the time trend in 5-year relative survival, using the R package periodR (version 1.0.6) [28,29]. We used multivariable Cox proportional hazards regression models to test the association between patient survival and residential area (urban [Winnipeg and Brandon] vs. rural), controlling for age at diagnosis, sex, year of diagnosis, and income quintile [30]. The proportional hazards (PH) assumption was examined using graphical and statistical approaches [30]. An interaction term with survival time was added to the model when a variable violated the PH assumption. Cox proportional hazards regression was undertaken using SAS 9.3 (SAS Institute, Cary, North Carolina).

## Results

Between 1984 and 2013, 511 cases (including 296 LL, 113 HL, and 100 NHL cases) were diagnosed in children and adolescents (Table 1). HL accounted for 45.6% of lymphomas in males and 60.6% in females. The sex ratios (M/F) were 1.4 for LL, 0.9 for HL, 1.7 for NHL (except Burkitt lymphomas [BL]), and 2.3 for BL. The median age at diagnosis ranged from 4 years (for LL) to 17 years (for mature B-cell lymphoma except BL).

**Table 1:** Lymphoid leukemia and lymphoma cases diagnosed among children and adolescents in Manitoba: 1984–2013.

Classification	Overall	Male	Female	M/F Ratio	Median age (years) at diagnosis
Total	511	287	224		
Lymphoid leukemia	296	171	125	1.4	4
Hodgkin lymphoma	113	53	60	0.9	17
Non-Hodgkin lymphoma (overall)	100	63	37	1.7	12
Non-Hodgkin lymphoma (except Burkitt lymphomas)	77	47	30	1.6	14
Precursor cell lymphomas	23	14	9	1.6	12
Mature B-cell lymphomas (except Burkitt lymphomas)	30	17	13	1.3	17
Mature T-cell and NK-cell lymphomas	13	8	S	1.6	13
Non-Hodgkin lymphomas, NOS	11	8	S	2.7	12
Burkitt lymphoma/leukemia	23	16	7	2.3	9
Unspecified lymphomas	S	0	S		8.5

NOS, not otherwise specified; S, suppressed for any values under 6.

Table 2 shows the age-standardized incidence rate (per million person-years) by sex and type. Age-standardized LL incidence rates were 34.0 (95% CI 28.9–39.1) and 26.2 (95% CI 21.5–30.7) in males and females, respectively. HL incidence rates were similar for males (10.5, 95% CI 7.7–13.3) and females (12.5, 95% CI 9.4–15.7). However, males had the higher incidence rates for overall NHL (12.5 vs 7.7), NHL except Burkitt lymphomas (9.3 vs. 6.3), and precursor cell lymphomas (2.8 vs 1.9) than females.

**Table 2:** Age-standardized lymphoid leukemia and lymphoma incidence rates (per million person-years) in children and adolescents in Manitoba, Canada: 1984–2013.

Classification	Male				Female			
	0–14 years		0–19 years		0–14 years		0–19 years	
	N	Rate (95% CI)	N	Rate (95% CI)	N	Rate (95% CI)	N	Rate (95% CI)
Lymphoid leukemia	155	41.5 (34.9–48.0)	171	34.0 (28.9–39.1)	117	32.9 (25.9–38.9)	125	26.2 (21.5–30.7)
Hodgkin lymphoma	19	5.1 (2.8–7.4)	53	10.5 (7.7–13.3)	13	3.7 (1.7–5.6)	60	12.5 (9.4–15.7)
Non-Hodgkin lymphoma (overall)	40	10.7 (7.4–14.0)	63	12.5 (9.3–15.4)	23	6.5 (3.8–9.1)	37	7.7 (5.2–10.2)
Non-Hodgkin lymphoma (except Burkitt lymphomas)	27	7.2 (4.5–9.9)	47	9.3 (6.7–12.0)	17	4.8 (2.5–7.1)	30	6.3 (4.0–8.5)
Precursor cell lymphomas	11	2.9 (1.2–4.7)	14	2.8 (1.3–4.2)	S	1.4 (0.2–2.6)	9	1.9 (0.6–3.1)
Mature B-cell lymphomas (except Burkitt lymphomas)	6	1.6 (0.3–2.9)	17	3.4 (1.8–4.9)	S	1.4 (0.2–2.6)	13	2.7 (1.2–4.2)
Mature T-cell and NK-cell lymphomas	6	1.6 (0.3–2.9)	8	1.6 (0.5–2.7)	S	1.4 (0.2–2.6)	S	1.0 (0.1–1.9)
Non-Hodgkin lymphomas, NOS	S	1.1 (0.0–2.1)	8	1.6 (0.5–2.7)	S	0.6 (0.0–1.3)	S	0.6 (0.0–1.3)
Burkitt lymphoma/leukemia	13	3.5 (1.9–5.4)	16	3.2 (1.6–4.7)	6	0.7 (0.3–3.0)	7	1.5 (0.4–2.5)
Unspecified lymphomas			0				S	0.6 (0.0–1.3)

N; number of case; NOS, not otherwise specified; S, suppressed for any values under 6.

**Fig 1:**
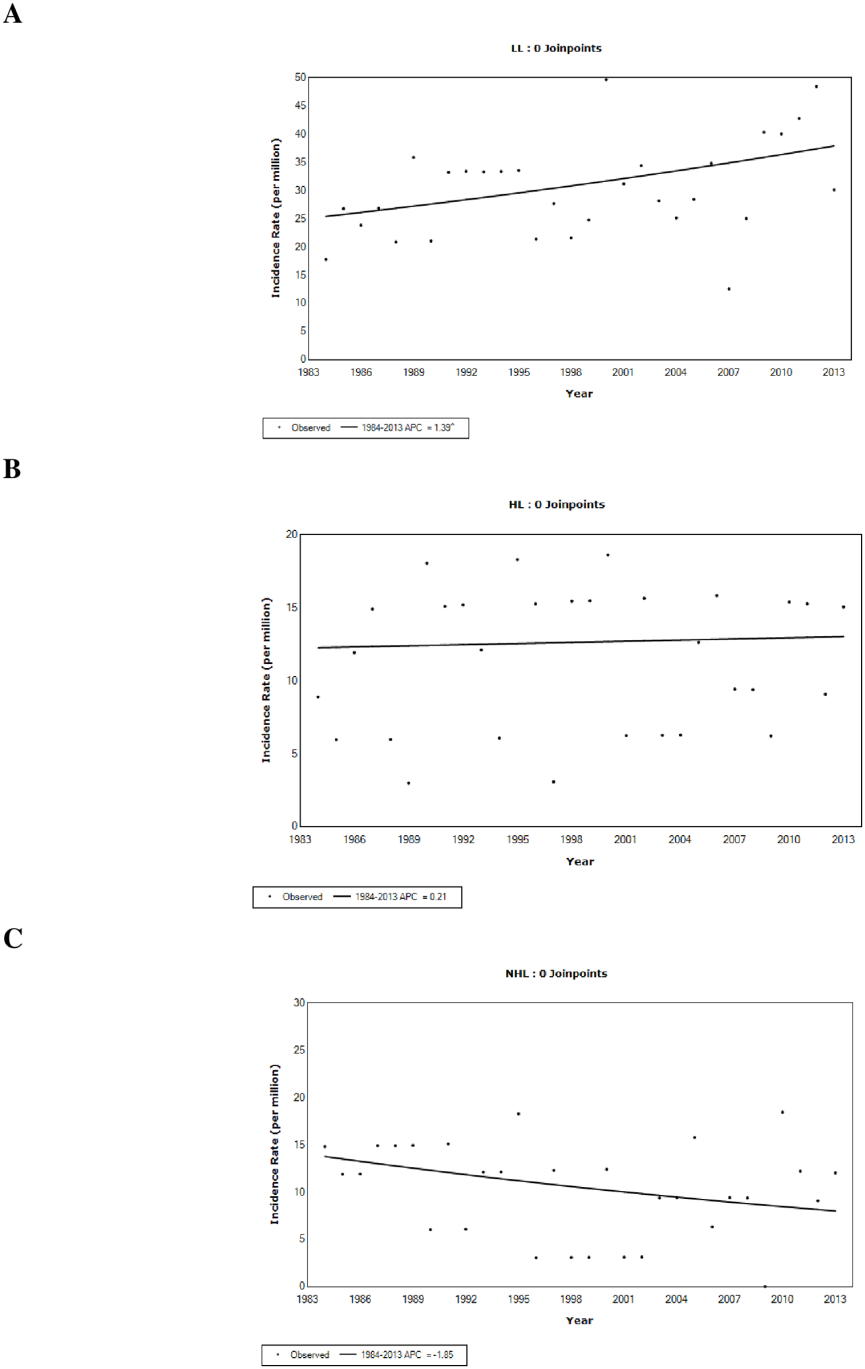
Time trends for age- and sex-standardized lymphoid leukemia (a), Hodgkin lymphoma (b), and non-Hodgkin lymphoma (c) incidence in children and adolescents in Manitoba, Canada: 1984–2013.

In the joinpoint analyses, zero joinpoint (i.e., straight line) was the best model to represent the time trends in LL, HL, and NHL incidence. While age- and sex-standardized LL incidence rates increased (APC = 1.4%, 95% CI 0.3% ~ 2.5%) over the past 3 decades (1984–2013), the rates for HL (APC = 0.2%, 95% CI -1.6% ~ 2.0%) and NHL (APC = -1.9%, 95% CI -5.9% ~ 2.3%) were relatively stable (Fig 1).

**Fig 2:**
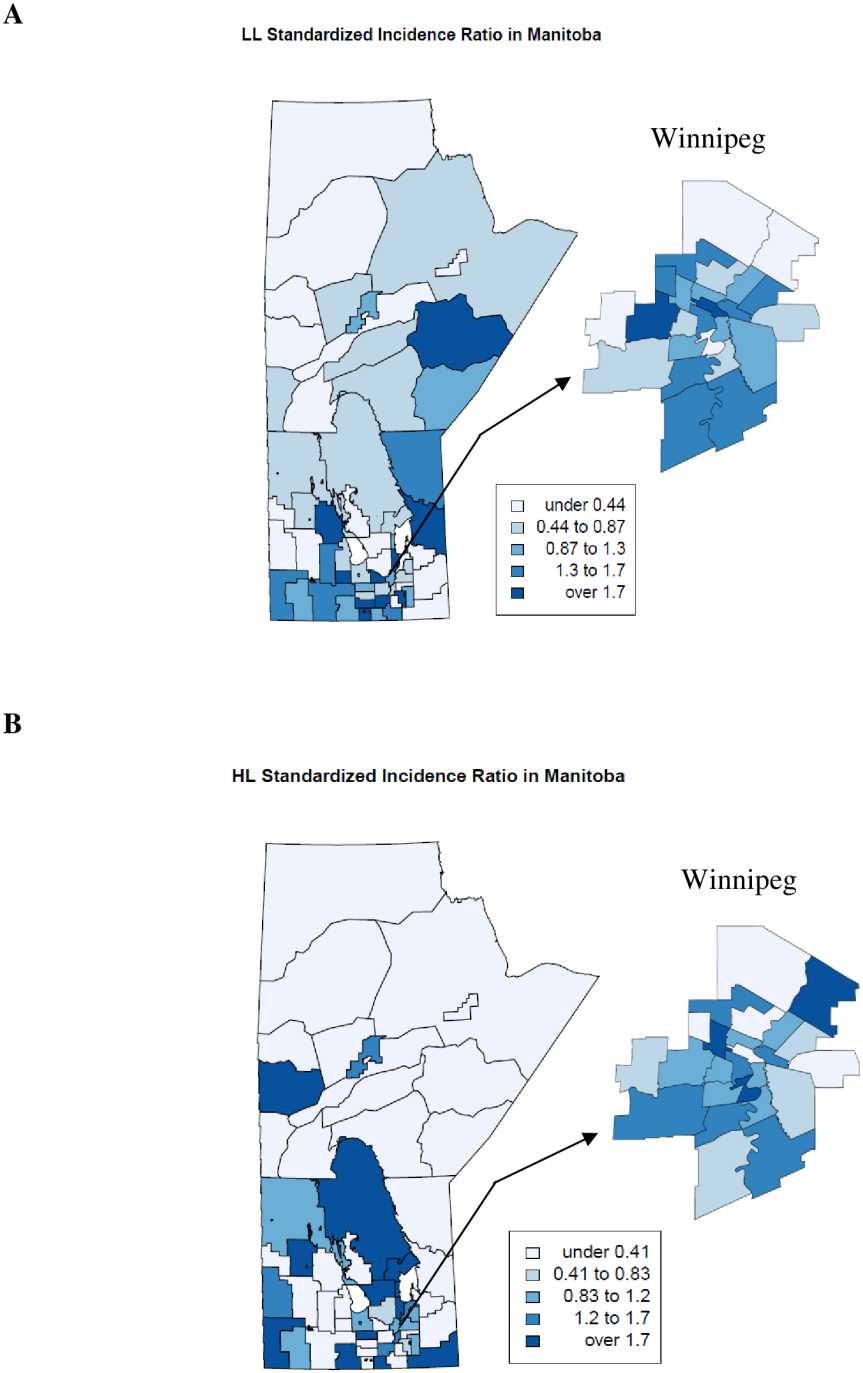
Geographical variations in lymphoid leukemia (LL), Hodgkin lymphoma (HL), and non-Hodgkin lymphoma (NHL) incidence in children and adolescents in Manitoba, Canada: 1984–2013.

Spatial analysis showed geographical variations in incidence rates of all types across the province and within Winnipeg. Outside Winnipeg, incidence rates for LL, HL, and NHL in Southern districts tended to be high, compared to the provincial average (Fig 2). Most likely clusters were identified in southern districts for all three types (S1 Fig). High incidence for NHL was observed in three northern districts, but no clustering was detected in this area. In Winnipeg, most likely clusters were found in Northwestern neighborhood clusters for LL, HL, and NHL (S1 Fig).

Ten-year and 15-year relative survival was slightly lower than 5-year relative survival for all types (S1 Table). Females generally had better survival than males. There was an trend of improved 5-year relative survival for all types of cancers, and the improvement was statistically significant for NHL in both males and females (Table 3). The greatest increase was observed for NHL in females, from 58.5% in 1984–1993 to 90.7% in 2004–2013. In the most recent period (2004–2013), 5-year relative survival was greater than 85% for all three types of cancers. In the Cox proportional hazards regression model, no statistically significant difference in patient survival was found between rural and urban areas, but the survival has improved overtime (S2 Table).

**Table 3:** Time trends in 5-year relative survival of lymphoid leukemia and lymphoma in children and adolescents in Manitoba, Canada.

Classification	Sex	1984–1993		1994–2003		2004–2013		Difference between 2004–2013 and 1984–1993	P value for time trend test *
		N	Relative Survival	N	Relative Survival	N	Relative Survival		
Lymphoid leukemia	Male	47	76.5 (7.1)	84	76.9 (5.7)	94	87.9 (4.3)	11.4	0.06
	Female	44	87.3 (6.1)	64	90.9 (4.4)	61	95.3 (3.3)	8.0	0.17
	Overall	91	81.4 (4.8)	144	83.1 (3.8)	155	90.9 (2.9)	9.5	0.03
Hodgkin lymphoma	Male	21	88.4 (11.1)	33	83.1 (7.8)	22	100	11.6	0.08
	Female	16	100	27	94.8 (5.4)	35	100	0	0.13
	Overall	37	94.2 (5.9)	60	88.3 (5.0)	57	100	5.8	0.02
Non-Hodgkin lymphoma	Male	27	74.4 (9.3)	26	86.7 (8.8)	28	85.9 (7.6)	11.5	0.04
	Female	14	58.5 (14.5)	16	64.4 (12.8)	14	90.7 (9.1)	32.2	0.03
	Overall	41	69.7 (7.8)	42	75.1 (8.2)	42	87.4 (5.9)	17.7	0.003

Note: numbers in parentheses are standard errors. NOS, not otherwise specified. N, number of case.

*, p value for time trend from Poisson regression model.

## Discussion

In this analysis, we estimated age-specific and standardised incidence rates for lymphoid malignancies in children and adolescents in Manitoba, Canada. Age-specific incidence patterns for LL, HL, and NHL are similar to that reported across the world [6]. The etiology of childhood hematological malignancies is poorly understood and there are only a few established risk factors [31]. The peak incidence rate for LL at age 0–4 years is consistent with the *in utero* origin hypothesis for childhood leukemia [32].

Age-standardized incidence rates for LL, HL, and NHL in Manitoba children and adolescents are in the range of those reported in Canada [33–37], United States [1], and other countries [8,38,39]. Countries around the world have different time trends in LL and lymphoma incidence rates [9]. We did not find published information on the time trend in LL incidence in Canadian children and adolescents. The present analysis showed that LL incidence rates in Manitoban children and adolescents increased 1.4% per year during 1984–2013. Overall leukemia incidence rates in Canadian boys increased by 0.8% annually during 1992–2010 [33], and this is largely driven by the increase (2.4% per year) during 1992–1999 [34]. Temporal trends in LL incidence varied by country: LL incidence rates increased 0.7% per year in children and adolescents in the United States (1975–2010) [1], 0.6% per year in children and 1.9% per year in adolescents in Europe (1978–1997) [40], but the increases in Western Australia (1960–2006) [41] and in Shanghai, China (1973–2005) [42] were not statistically significant. Rising incidence could be attributed to increased exposure to risk factors (e.g., environmental exposures) and/or diagnostic improvement. Further research is warranted to explore changes in risk factors for childhood LL [31] in the province and their possible contributions to the rise in incidence.

While HL incidence rates decreased 0.7% annually in children in USA (1975–2010) [1], the rates increased 1% per year among children aged 10–14 years and 3.5% per year among adolescents in Europe during 1978–1997 [43]. Like in this analysis, a previous study found no increase in incidence rate for NHL in children of Canada [35]. But NHL incidence rates increased 0.9% per year in children and 1.7% per year in adolescents during 1978–1997 in Europe [44] and increased 1.1% per year during 1975–2010 in USA [1]. Rising HL incidence when NHL incidence is declining have been attributed to diagnostic misclassification of NHL as HL [45]. When HL and NHL were combined, incidence rates were stable in Canada (1992–2010) [33], Australia (1983–2006) [46], and China (1973–2005) [42]. Age-standardized incidence rates for BL (one of the most common NHL subtypes] in males and females, were higher than that reported in a recent analysis of BL cases registered with the International Agency for Research on Cancer [47]. The risk of endemic BL in Africa is related to malaria, Epstein-Barr virus, and human immunodeficiency virus infections, but little is known about the etiology of sporadic BL [48]. Further research is needed to explain the high BL incidence in this province.

The observed spatial variations in LL, HL, and NHL incidence in this study and others [7,49,50] indicate variations in exposure to genetic and environmental risk factors. Genetic factors account for a small proportion of childhood cancers and environmental factors may play a greater role [51]. Point source of known carcinogens such as radiation was associated with increased leukemia risk in children living nearby [52]. The higher incidence in southern Manitoba, where socioeconomic status (SES) is better than that in other areas, is consistent with the finding from some of previous studies in other jurisdictions. Communities with high area-based measure of SES had higher childhood leukemia incidence than those with lower SES [53–55], but the association was not observed in other ecological studies [56,57]. On the contrary, many studies with individual-level SES measures found an inverse association between SES and childhood leukemia incidence [58,59]. Overall, findings regarding the association between SES and childhood leukemia risk are mixed. The heterogeneity may be due to differences in place and calendar time when the studies were undertaken and in SES measures used [58,59]. Fewer studies have examined the association between SES and lymphoma (HL and NHL) incidence [56,60]. Geographic variations remained after adjusting for average household income, indicating other area-related factors (e.g., environmental exposures) might play a role. However, without data on the geographical difference in risk factor exposures, it is unclear whether and to what extent the distributions of these risk factors have contributed to the geographical disparities. The interpretation of the higher NHL incidence in remote Northern regions may require caution due to the very small population size in these areas. It is unlikely that environmental exposures played a role, but infections and diet might.

Similar to those reported in other jurisdictions [1,8,38,39], the vast majority of children and adolescents have survived for longer than 5 years after leukemia/lymphoma diagnosis. Survival of patients with LL, HL, and NHL has increased over time in Manitoba and other areas [1,8,38,39], most likely reflecting the treatment improvement for leukemia and lymphoma patients. As treatment advances, the number of child and adolescent leukemia and lymphoma survivors will continue to increase. Appropriate therapy-related and risk-based long-term follow-up is important to monitor late effects (e.g., pulmonary dysfunction, cardiac disease, infertility, and second malignant neoplasms) of treatments and to improve those patients’ quality of life [61].

Previous studies have shown geographical disparities in childhood leukemia and lymphoma survival related to remoteness and SES [7,13,62,63]. But no statistically significant difference in survivals was found between patients living in rural and urban areas in the present study, which may reflect improved access due to the implementation in 1978 of the Manitoba Community Cancer Programs Network (MCCPN) program, a provincial program operated by CancerCare Manitoba (CCMB) to provide rural cancer patients care and treatment in or near their own communities [64]. The MCCPN program has 16 outpatient units located in community hospitals across the province, avoiding nine million kilometers of travel to and from urban centers for patients and their families each year [65].

This study has some limitations. Reporting delay [66], the time elapsed before a diagnosed cancer case is reported to a cancer registry, was not used to adjust incidence rate calculations as delay adjustment data are not available for this population. The delay primarily affects the estimation of incidence rates in the most recent 1 to 3 years (2011–2013 in this case) and the actual incidence rates in these years might have been underestimated. Spatial analysis was based on the most recent geographical boundary, but postal code and boundary went through several changes during the study period. In the Cox regression model, we combined the three cancer types in order to generate more robust analysis (higher number of cases). However, the three types of lymphoid cancers are heterogeneous. The prognosis of lymphoid malignancy patients is determined by many factors other than those tested in the analysis. LL patients are at different levels of risk associated with phenotype, age at diagnosis, initial white blood cell count, and other prognostic factors, but we did not have sufficient data and sample size for subgroup analysis. The mortality rate was not reported because of the extremely low number of death cases in children and adolescents in the province (28 LL deaths, 1 HL death, and 25 NHL deaths during 1982–2014). Other studies demonstrated that LL, HL, and NHL were cancer types with the most significant mortality declines in children and adolescents over the past three decades [1,67].

The analysis showed an increase in LL incidence but not in the other two types in the province of Manitoba, Canada. Geographical variation and clustering in leukemia and lymphoma incidence might be due to differences in risk factors, but further research is needed to identify possible factors and their relations to leukemia and lymphoma risk. The observed survival improvement likely reflects advances in leukemia and lymphoma treatments for children and adolescents.

## Supporting information

S1 Fig Most likely clusters for lymphoid leukemia (LL), Hodgkin lymphoma (HL), and non-Hodgkin lymphoma (NHL) incidence in children and adolescents in Manitoba, Canada: 1984–2013. (DOCX)

S1 Table Observed and relative survival by sex in children and adolescents with lymphoid leukemia or lymphoma in Manitoba, Canada, 1984–2013. (DOCX)

S2 Table Hazard ratios (HRs) and 95% confidence intervals (CIs) from Cox regression model. (DOCX)

## Abbreviations

95% CI: 95% confidence interval
APC: annual percentage change
BL: Burkitt lymphomas
HL: Hodgkin lymphoma
HR: hazard ratio
ICCC-3: International Classification of Childhood Cancer, 3rd Edition
ICD: International Classification of Diseases
LL: lymphoid leukemia
NHL: non-Hodgkin lymphoma
RS: relative survival
