# Supplementary material for: Time and spatial trends in lymphoid leukemia and lymphoma incidence and survival among children and adolescents in Manitoba, Canada: 1984-2013
Source: PLoS One. 2017 Apr 21;12(4):e0175701. doi: 10.1371/journal.pone.0175701 (PMC5400229; doi:10.1371/journal.pone.0175701)
Supplement: S1 Fig — (DOCX) [file pone.0175701.s001.docx]

S2 Fig. Most likely clusters for lymphoid leukemia (LL), Hodgkin lymphoma (HL), and non-Hodgkin lymphoma (NHL) incidence in children and adolescents in Manitoba, Canada: 1984-2013

**A**

Winnipeg

**Most likely cluster for childhood LL incidence in Manitoba**

**B**

Winnipeg

**Most likely cluster for childhood HL incidence in Manitoba**

**C**

**Most likely cluster for childhood NHL incidence in Manitoba**

Winnipeg
