# Supplementary material for: Time and spatial trends in lymphoid leukemia and lymphoma incidence and survival among children and adolescents in Manitoba, Canada: 1984-2013
Source: PLoS One. 2017 Apr 21;12(4):e0175701. doi: 10.1371/journal.pone.0175701 (PMC5400229; doi:10.1371/journal.pone.0175701)
Supplement: S1 Table — (DOCX) [file pone.0175701.s002.docx]

S1 Table. Relative survival by sex in children and adolescents with lymphoid leukemia or lymphoma in Manitoba, Canada, 1984-2013

| Classification | Sex | N | 5-year | 10-year | 15-year |
| --- | --- | --- | --- | --- | --- |
| Lymphocytic leukemia | Male | 171 | 81.0 (3.2) | 77.7 (3.5) | 75.3 (3.8) |
|  | Female | 125 | 91.7 (2.6) | 91.8 (2.6) | 92.0 (2.6) |
|  | Overall | 296 | 85.6 (2.2) | 83.8 (2.3) | 82.7 (2.5) |
|  |  |  |  |  |  |
| Hodgkin Lymphoma | Male | 53 | 89.7 (4.4) | 89.6 (4.4) | 90.0 (4.4) |
|  | Female | 60 | 98.3 (2.1) | 94.3 (3.6) | 94.7 (3.7) |
|  | Overall | 113 | 94.1 (2.4) | 92.2 (2.8) | 92.5 (2.8) |
|  |  |  |  |  |  |
| Non-Hodgkin lymphoma | Male | 63 | 81.5 (5.1) | 81.7 (5.1) | 79.2 (5.6) |
|  | Female | 37 | 69.7 (7.7) | 70.0 (7.7) | 70.2 (7.7) |
|  | Overall | 100 | 77.1 (4.3) | 77.7 (3.5) | 75.7 (4.6) |

Note: Numbers in parentheses are standard errors. N, number of cases.
