# Supplementary material for: Time and spatial trends in lymphoid leukemia and lymphoma incidence and survival among children and adolescents in Manitoba, Canada: 1984-2013
Source: PLoS One. 2017 Apr 21;12(4):e0175701. doi: 10.1371/journal.pone.0175701 (PMC5400229; doi:10.1371/journal.pone.0175701)
Supplement: S2 Table — (DOCX) [file pone.0175701.s003.docx]

S2 Table. Hazard ratios (HRs) and 95% confidence intervals (CIs) from Cox regression model

| Variable |  | HR | 95% CI |
| --- | --- | --- | --- |
| Age (years)^#^ | <=4 | 1.08 | 0.52-2.19 |
|  | 5-9 | 0.59 | 0.27-1.28 |
|  | 10-14 | 0.90 | 0.47-1.72 |
|  | 15-19 | Reference |  |
| Sex | Male | 1.37 | 0.86-2.17 |
|  | Female | Reference |  |
| Residential area | Rural | 1.48 | 0.95-2.31 |
|  | Urban^*^ | Reference |  |
| Income quintile^±^ | Lowest (Q1) | 1.11 | 0.53-2.34 |
|  | Q2 | 1.28 | 0.64-2.53 |
|  | Q3 | 1.00 | 0.47-2.13 |
|  | Q4 | 1.17 | 0.58-2.34 |
|  | Highest (Q5) | Reference |  |
| Year of diagnosis | 1984-1993 | 2.18 | 1.10-4.30 |
|  | 1994-2003 | 1.96 | 0.99-3.90 |
|  | 2004-2013 | Reference |  |

^#^ An interaction with survival time was added for this variable; ^*^ Including cities Winnipeg and Brandon; ^±^ Based on average neighborhood household income.
